# Supplementary material for: Mapping effector genes at lupus GWAS loci using promoter Capture-C in follicular helper T cells
Source: Nat Commun. 2020 Jul 3;11:3294. doi: 10.1038/s41467-020-17089-5 (PMC7335045; doi:10.1038/s41467-020-17089-5)
Supplement: Supplementary file 1 — Supplementary Information [file 41467_2020_17089_MOESM1_ESM.pdf]

Supplementary Information

"Mapping effector genes at lupus GWAS loci using promoter Capture-C in follicular helper T cells," Su et. al.

SUPPLEMENTARY FIGURE 1

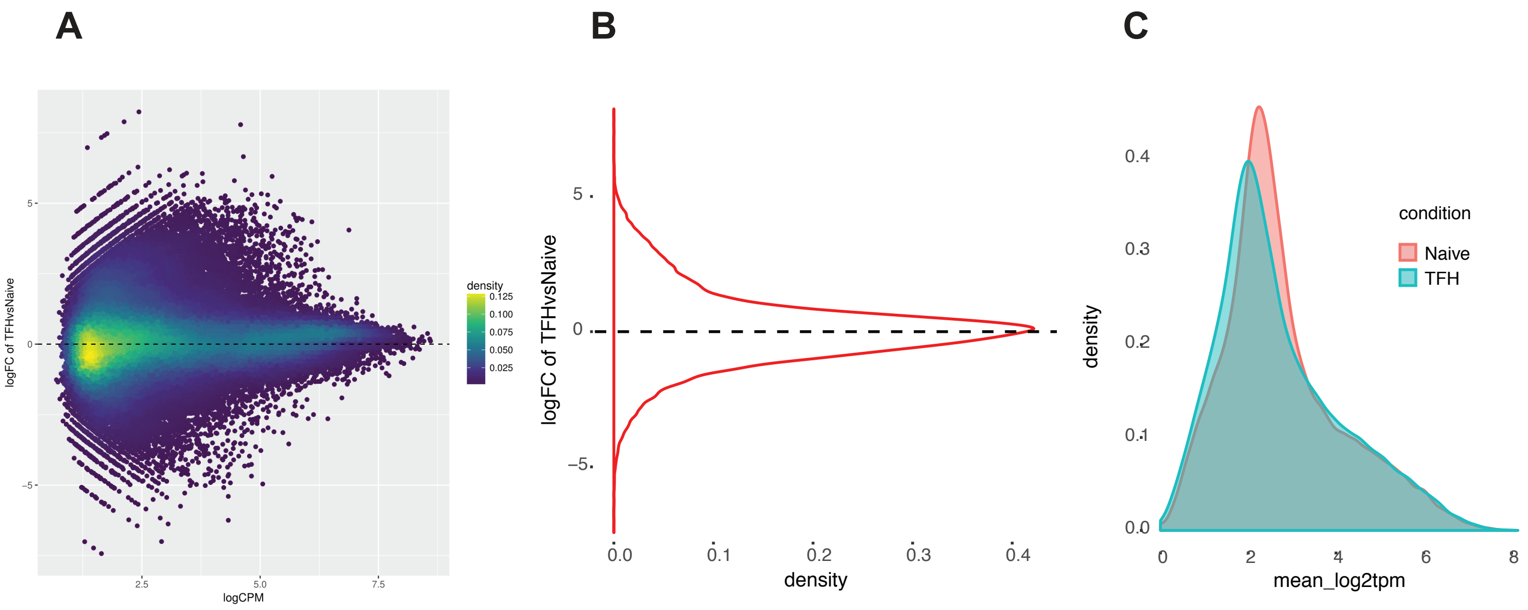

SUPPLEMENTARY FIGURE 1. TFH and naive T cells show comparable genomic accessibility. Overall log2 fold changes in reference OCR accessibility (CPM) in TFH compared to naive T cells represented by density plot (a) or distribution plot (b). c. The accessibility signal was normalized by the counts per million method and mean p values across three replicates were used for comparison between TFH and naive T cells.

SUPPLEMENTARY FIGURE 2

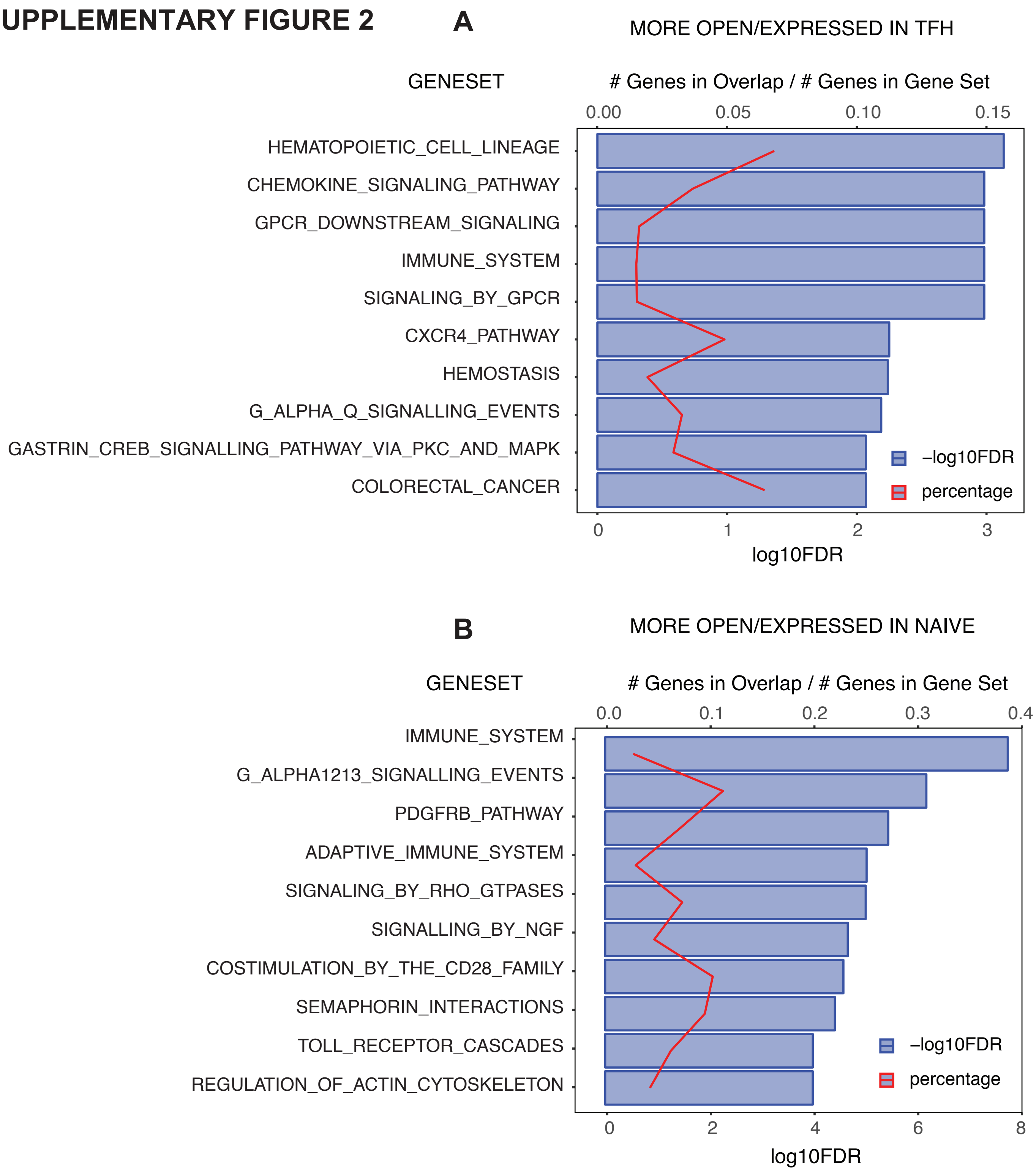

SUPPLEMENTARY FIGURE 2. Canonical pathway enrichment for genes with accessible SLE SNPs in their promoters. The log2FDR (blue) and gene ratios (red) for the top 10 enriched Ingenuity canonical pathways is shown for TFH (a) and naive (b) cells.

SUPPLEMENTARY FIGURE 3 A-E

A *IL21*

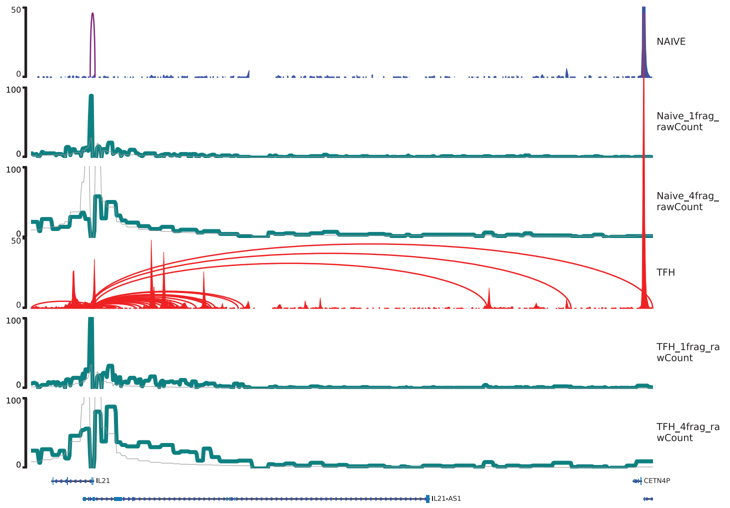

B *IFNG*

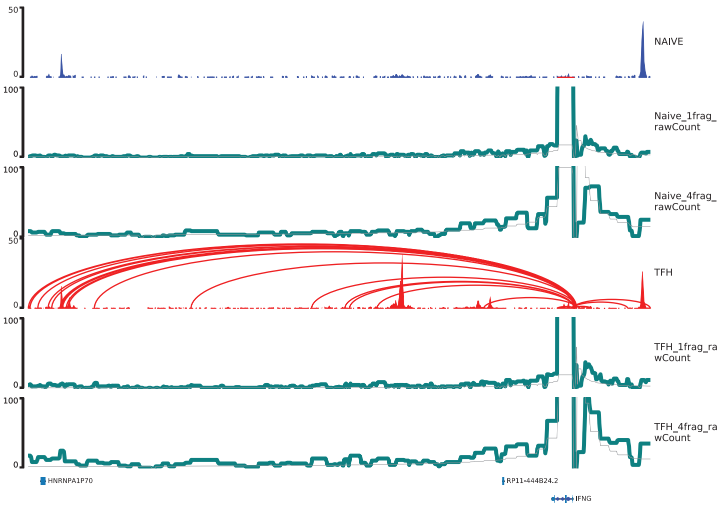

C *CD28*

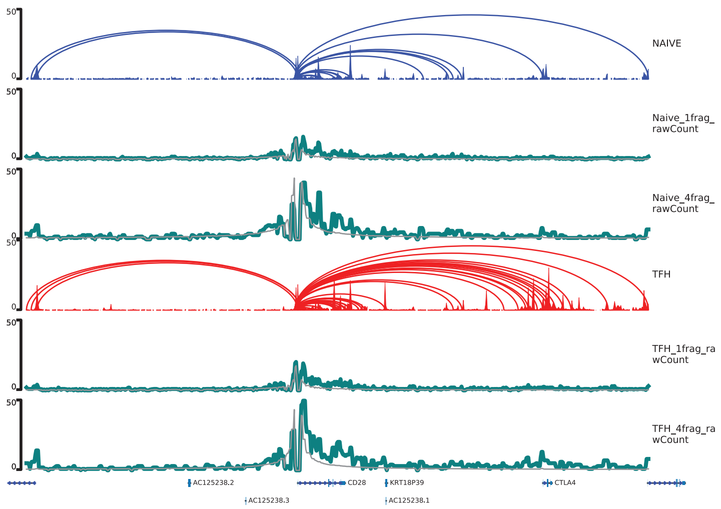

D *CTLA4*

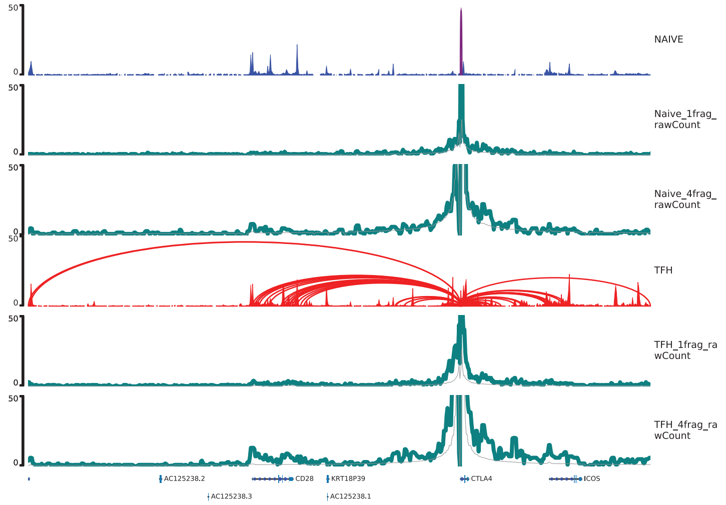

E *ICOS*

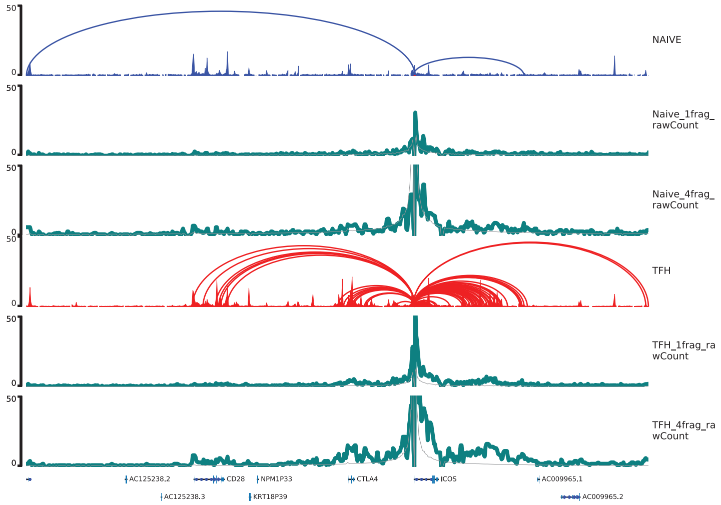

SUPPLEMENTARY FIGURE 3 F-N

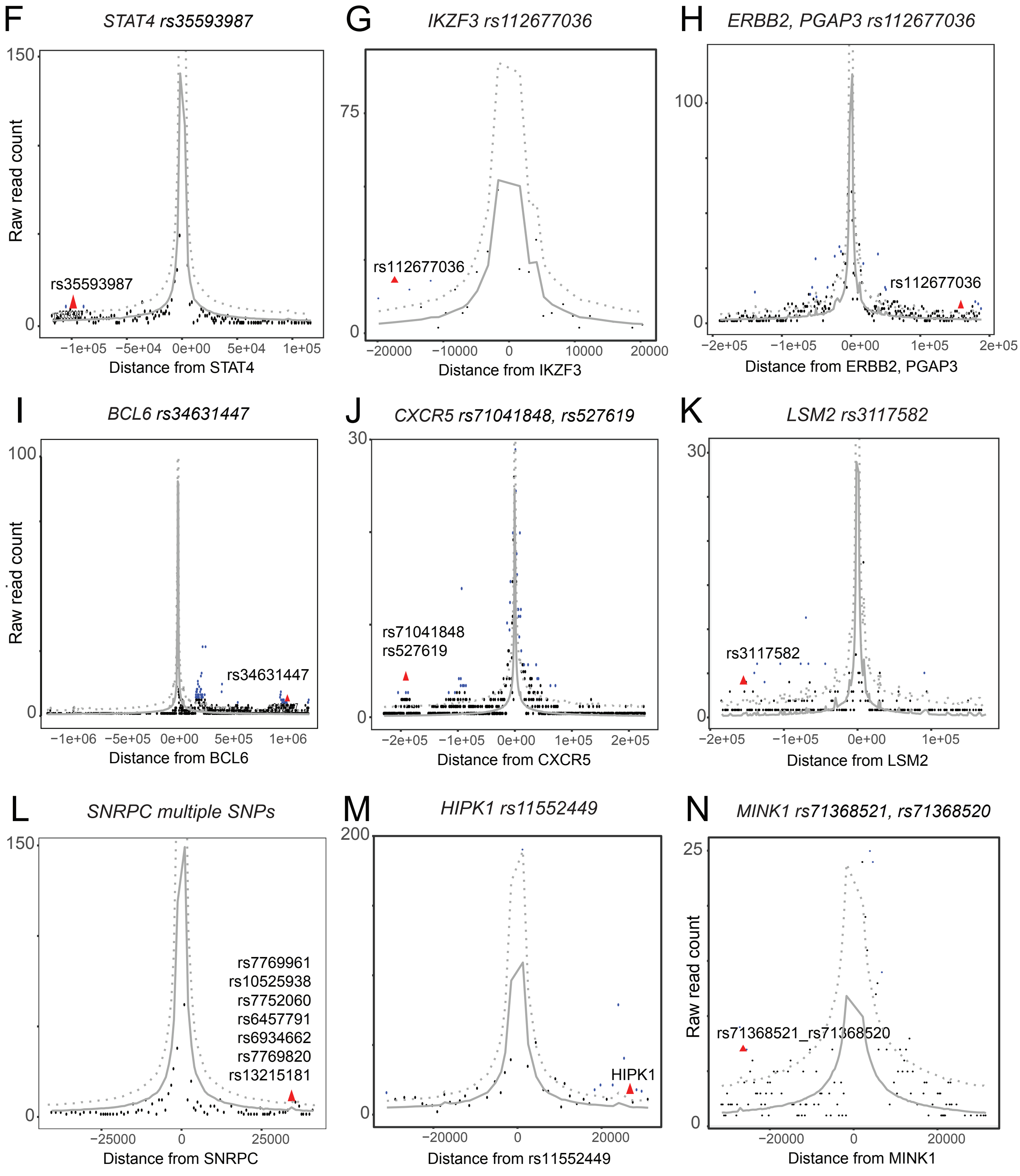

SUPPLEMENTARY FIGURE 3. Raw paired read count data supporting significant CHiCAGO promoter interaction calls. A-E. Fragment interactions with gene promoter baits of IL21 (A), IFNG (B), CD28 (C), CTLA4 (D) and ICOS (E) in naïve and TFH. The arc tracks represent the significant interaction (Chicago score > 5) between promoter bait and promoter interacting regions (PIRs). The interactions from 1frag and 4frag resolution were merged while only the higher 1frag resolution was used if interactions were called in both resolutions. The raw read pair count (green line) and the expected level of Brownian collision background (grey line) were plotted per bait at both 1frag and 4frag resolution. F-N. Raw paired read count data supporting significant CHiCAGO promoter-SNP interaction calls for STAT4 (F), IKFZ3 (G), ERBB2 (H), BCL6 (I), CXCR5 (J), LSM2 (K), SNRPC(L), HIPK1 (M) and MINK1 (N) in TFH. Centered at promoter bait, the raw read pair count for each PIR was plotted as a dot within the distance between bait and SNP-containing PIR at the resolution of this interaction detected. The points representing significant interactions (Chicago score > 5) is colored blue and red triangle indicates the significant interaction between bait and SNP(s)-containing PIR. The expected level of Brownian collision background (solid line) and upper limit of 95% confidence of Brownian background (dashed line) were plotted per bait at corresponding resolution.

SUPPLEMENTARY FIGURE 4

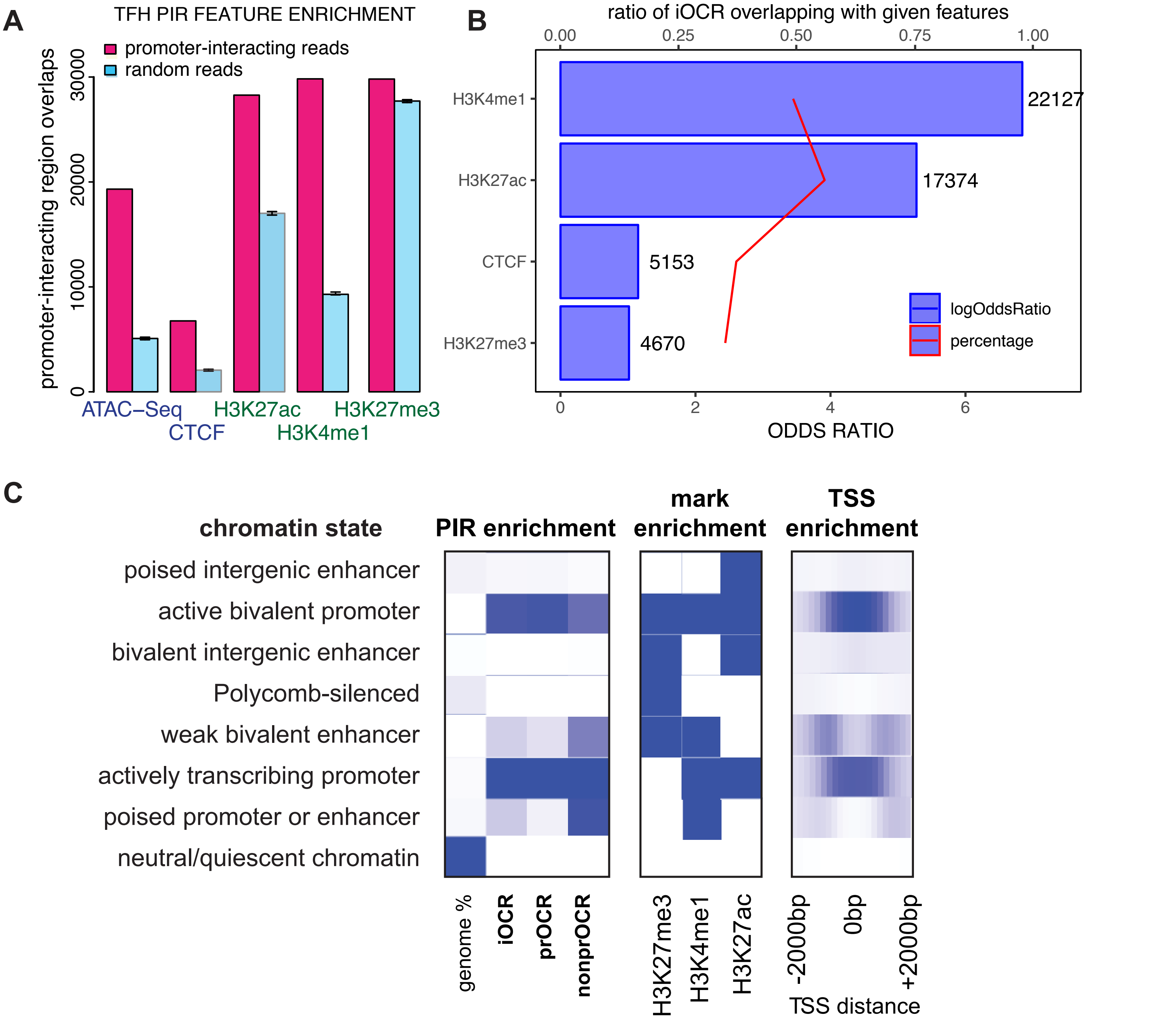

SUPPLEMENTARY FIGURE 4. Enrichment of chromatin signatures at promoter interacting regions in TFH cells. a. PIR enrichment for genomic features compared with distance-matched random regions in TFH cells. Error bars show 95% CI across 100 draws of non-significant interactions. b. Feature enrichment of promoter-interacting OCR (iOCR) compared to a random sample of non-promoter-interacting OCR in TFH. c. Enrichment of iOCR within chromHMM-defined chromatin states and TSS neighborhood in TFH. Roadmap Epigenomics 8-state models (middle panel) were defined on the basis of 3 histone modifications (H3K4me1, H3K4me3, H3K27me3, H3K27ac and H3K36me3). Blue color intensity represents the probability of observing the mark in the state. The heatmap to the left of the emission parameters displays the overlap fold enrichment for different categories of iOCR, while the heatmap to the right shows the fold enrichment for each state within 2 kb around a set of TSS. Blue color intensity represents fold enrichment.

SUPPLEMENTARY FIGURE 5

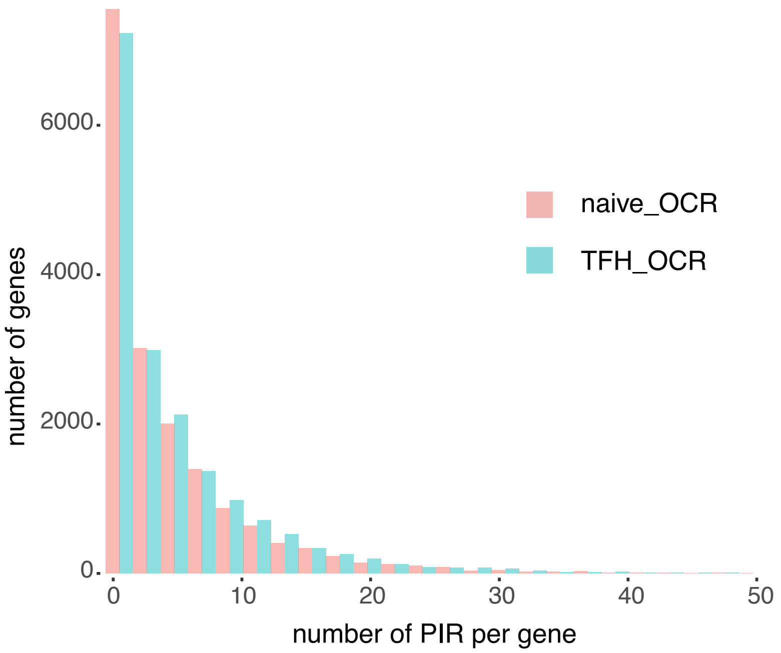

SUPPLEMENTARY FIGURE 5. Distribution of promoter-interacting OCR per gene in naïve T and TFH cells. The number of promoter-interacting OCR per gene is plotted for both naïve T (red) and TFH (blue) cells.

SUPPLEMENTARY FIGURE 6

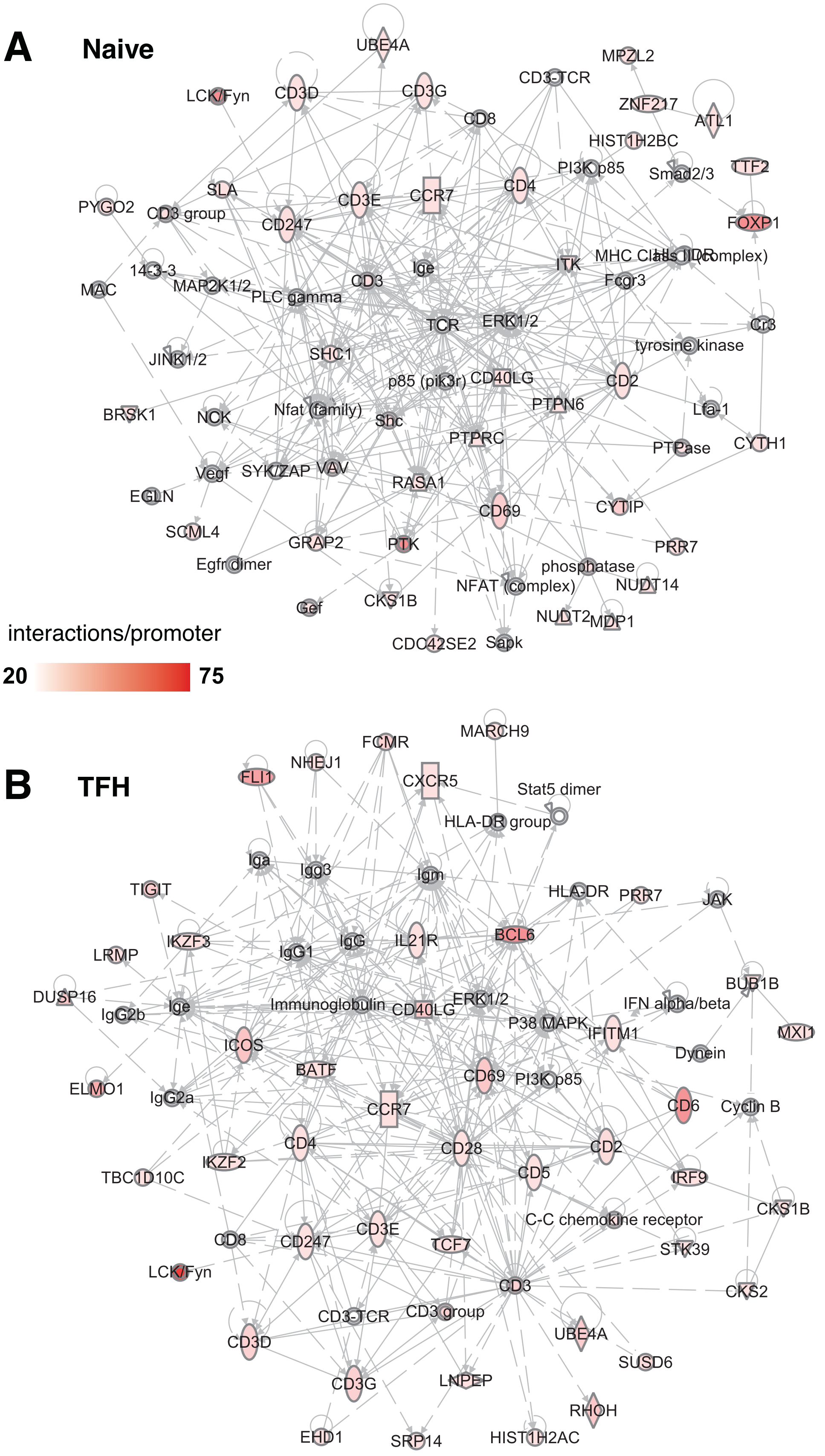

SUPPLEMENTARY FIGURE 6. Immune networks enriched among SLE SNP connectome implicated gene sets. The top 3 merged immune networks in naïve (a) and TFH (b) are depicted. Red color intensity represents the number of interactions detected per promoter for each gene in the network.

SUPPLEMENTARY FIGURE 7

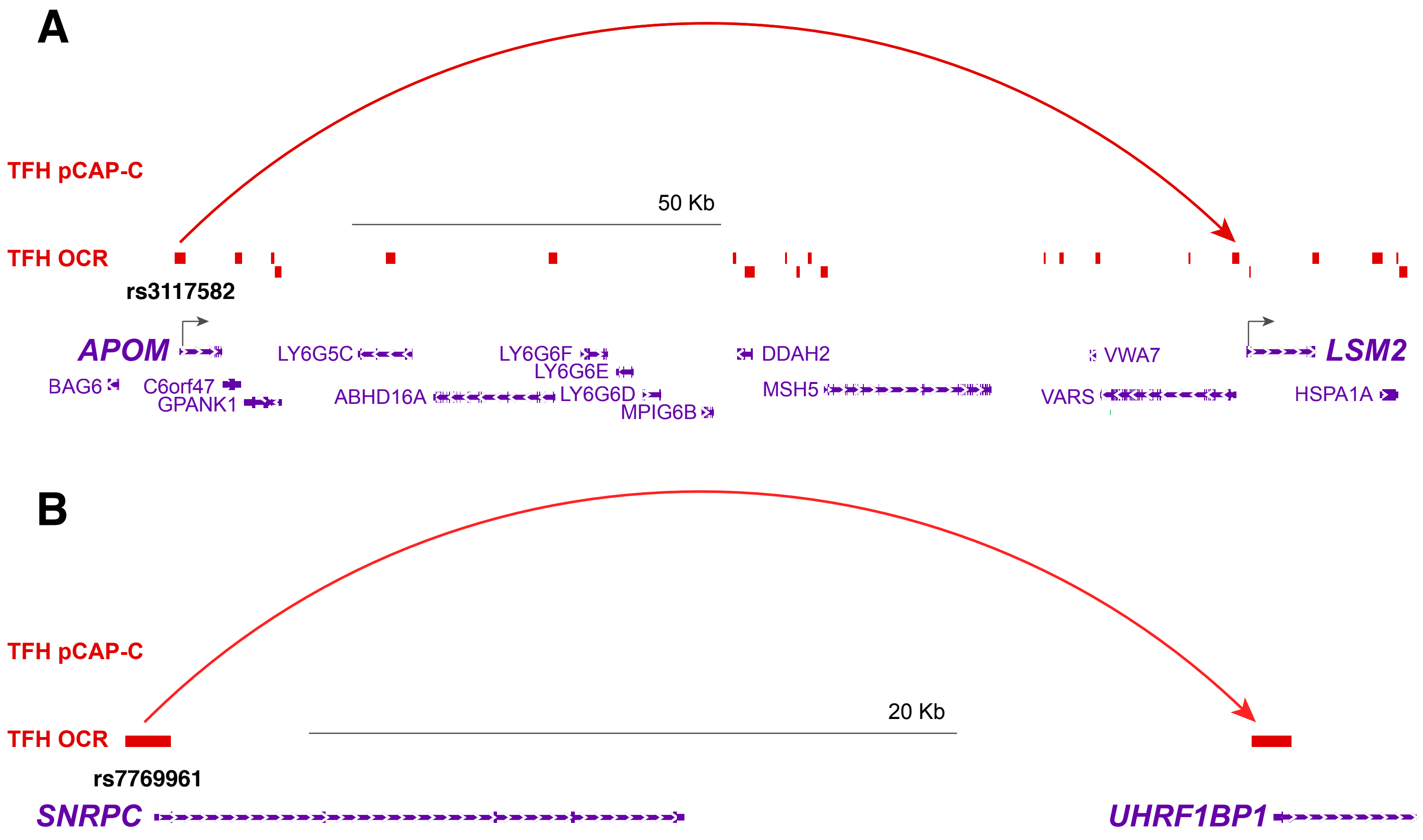

SUPPLEMENTARY FIGURE 7. Interaction of open SLE variants with genes encoding nuclear proteins targeted by autoantibodies in SLE patients. a. The accessible SNP rs3117582 at the promoter of APOM physically interacts with the LSM2 promoter. b. The accessible SNP rs7769961 at the SNRPC promoter physically interacts with the UHRF1BP1 promoter.

SUPPLEMENTARY FIGURE 8

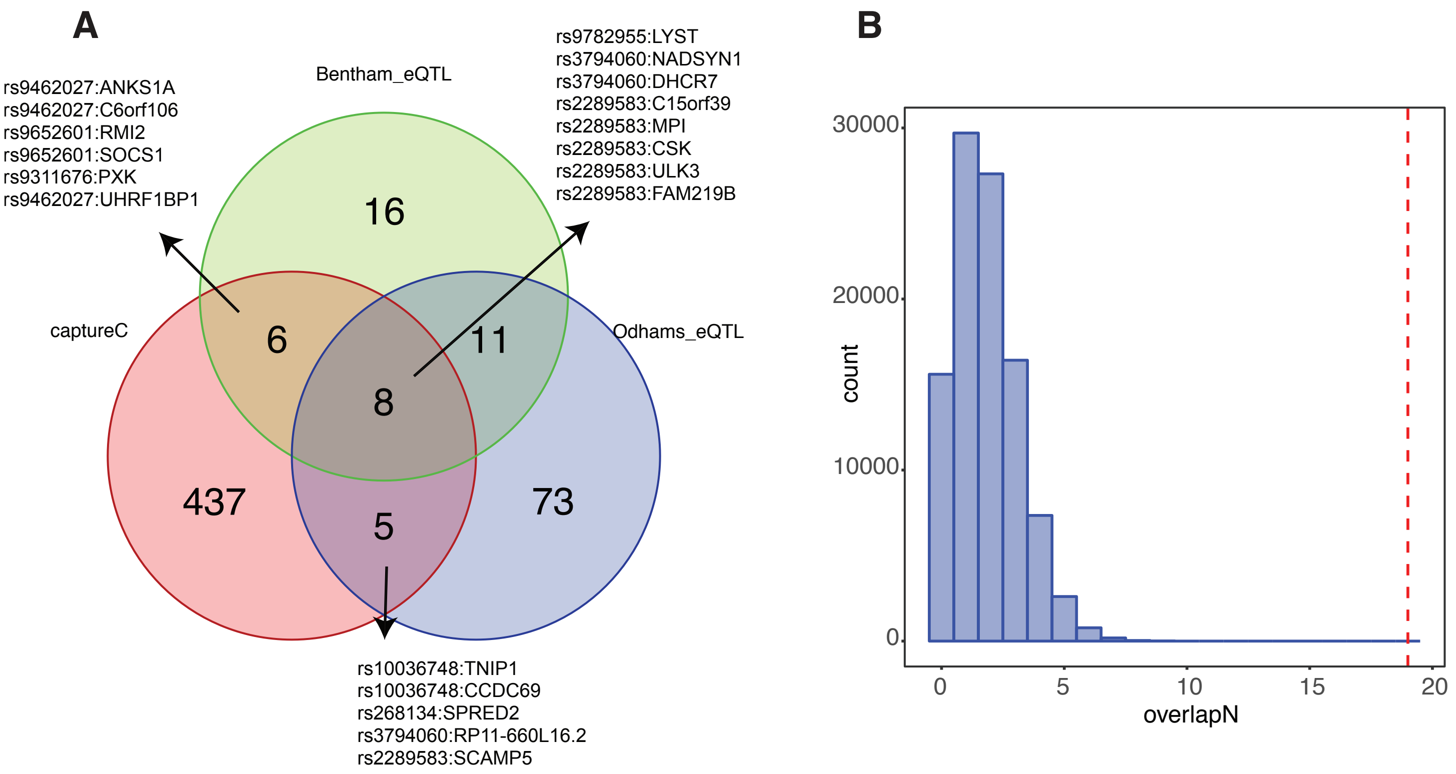

SUPPLEMENTARY FIGURE 8. Comparison of SLE SNP-gene associations obtained by promoter-open chromatin connectomes vs. eQTL studies. a. Comparison of sentinel SNP-gene pairs implicated by the promoter-open chromatin connectomes in this study vs. sentinel SNP-gene pairs statistically associated in two SLE eQTL studies7,29. SNP-gene pairs shared by each group are detailed. b. An empirical distribution hypothesis testing approach (see methods) was used to compare the observed overlap between SLE variant-connected genes and SLE eQTL genes (19) and the overlap expected at random (1).

SUPPLEMENTARY FIGURE 9

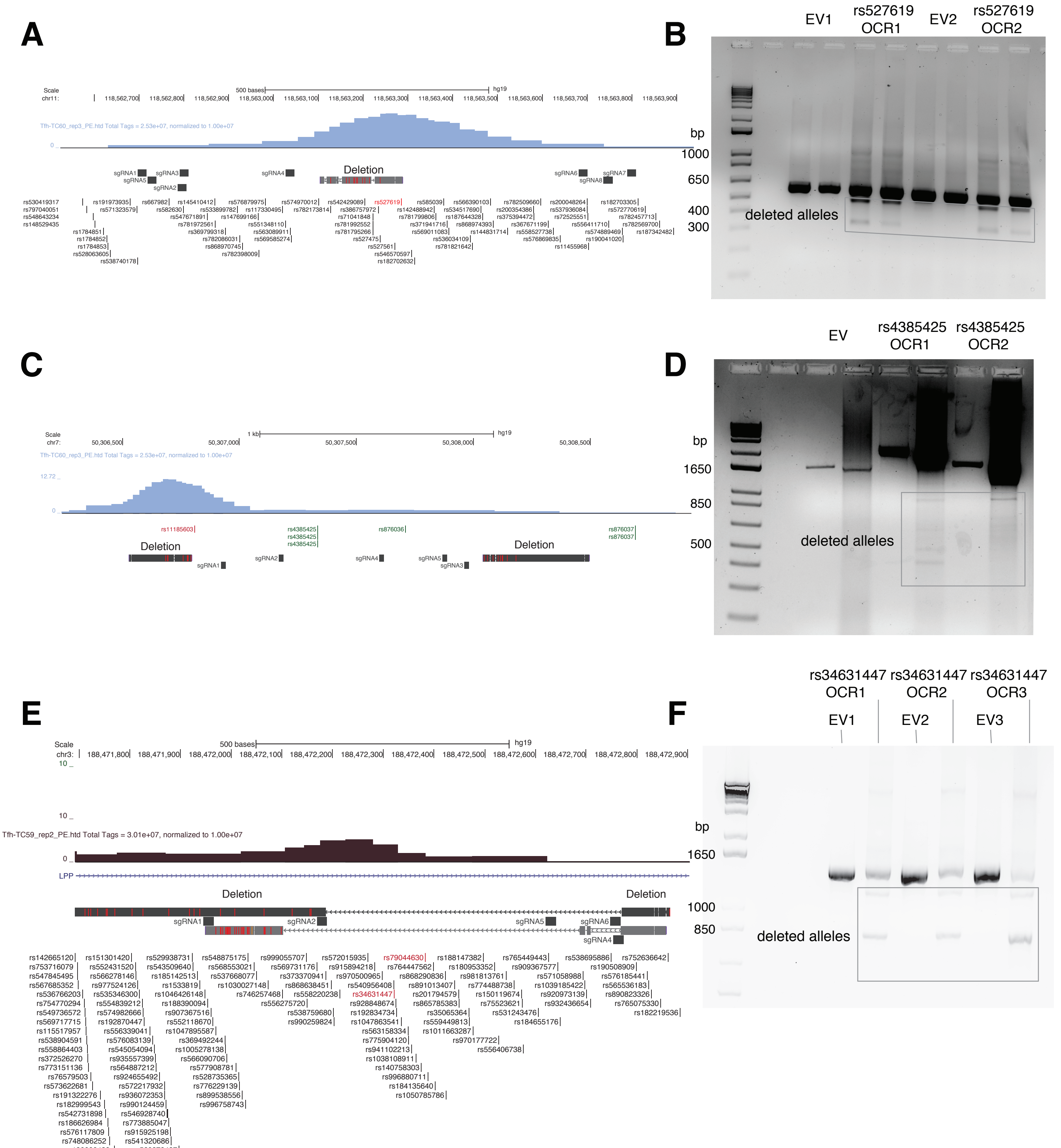

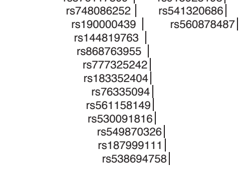

SUPPLEMENTARY FIGURE 9. CRISPR/CAS9-based editing of accessible genomic regions containing SLE GWAS proxy SNP in Jurkat T cells. a, UCSC genome browser track displaying intergenic TFH OCR near the TREH gene harboring the rs527619 and rs71041848 proxies to the rs4639966 SLE sentinel SNP that interacts with CXCR5 (chr11:118,563,185-118,563,321). Eight sgRNAs flanking the OCR and Sanger sequencing identifying several deletions present within the OCR are depicted. b, Electrophoresis gel analysis of PCR amplified regions encompassing the targeted region showing two distinct deletions present at 500bp and 350bp. c, Genomic region surrounding the TFH OCR containing the rs4385425 SNP proxy to the sentinel SLE SNP rs11185603 (chr7:50307234-50307447) that interacts with IKZF1, showing sgRNAs and deletions detected in targeted Jurkat lines. Note that this OCR was called by HOMER, but not by MACS2. d, Electrophoresis gel analysis detects three different deletions at 900bp, 400bp and 350bp. e, Intronic OCR (chr3:188,472,234-188,472,390) in the LPP locus harboring the rs34631447 and rs79044630 SNPs proxy to sentinel rs6762714 SLE SNP and found connected to BCL6. This region was targeted with five total sgRNAs surrounding the OCR and Sanger sequencing showed two distinct deletions. f, Electrophoresis gel analysis detects 1200bp and 821bp deletions. All experiments were performed in three biological replicates.

SUPPLEMENTARY FIGURE 10

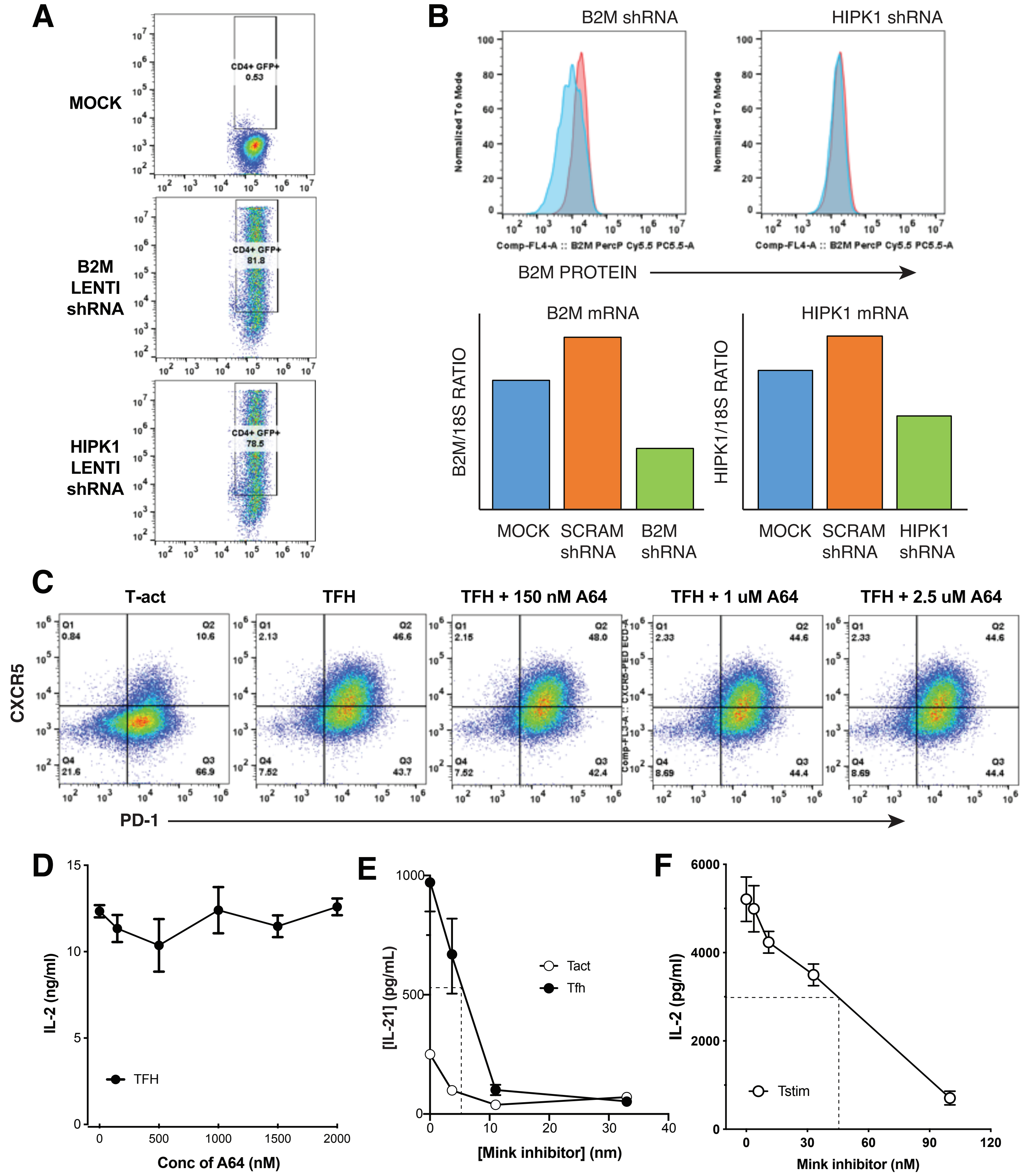

SUPPLEMENTARY FIGURE 10. Promoter-variant connectome-guided targeting of novel kinases for modulation of primary human TFH function. a, Lentiviral delivery of B2M shRNA and HIPK1 shRNA into in vitro differentiated TFH as assessed by GFP fluorescence by flow cytometry. b, Assessment of shRNA-mediated knock-down of B2M and HIPK1 in TFH by flow cytometry and qRT-PCR. Red histograms are TFH transduced with scrambled control shRNA, and blue histograms depict TFH transduced with specific B2M (left panel) or HIPK1 (right panel) shRNA. c, Effect of HIPK inhibitory drug treatment on TFH differentiation in vitro as measured by co-induction of PD-1 and CXCR5. d, The HIPK inhibitory drug A64 does not affect IL-2 secretion by TFH cells as measured by ELISA. e. A MINK inhibitory drug causes dose-dependent inhibition of IL-21 production by Tact and TFH cells with an ED50 of ~5.0 nM. f, A MINK inhibitory drug inhibits IL-2 secretion by activated T cells with an ED50 of ~50 nM. Data in d-f are mean +/- s.d. All data are representative of 3 replicate experiments.

SUPPLEMENTARY FIGURE 11

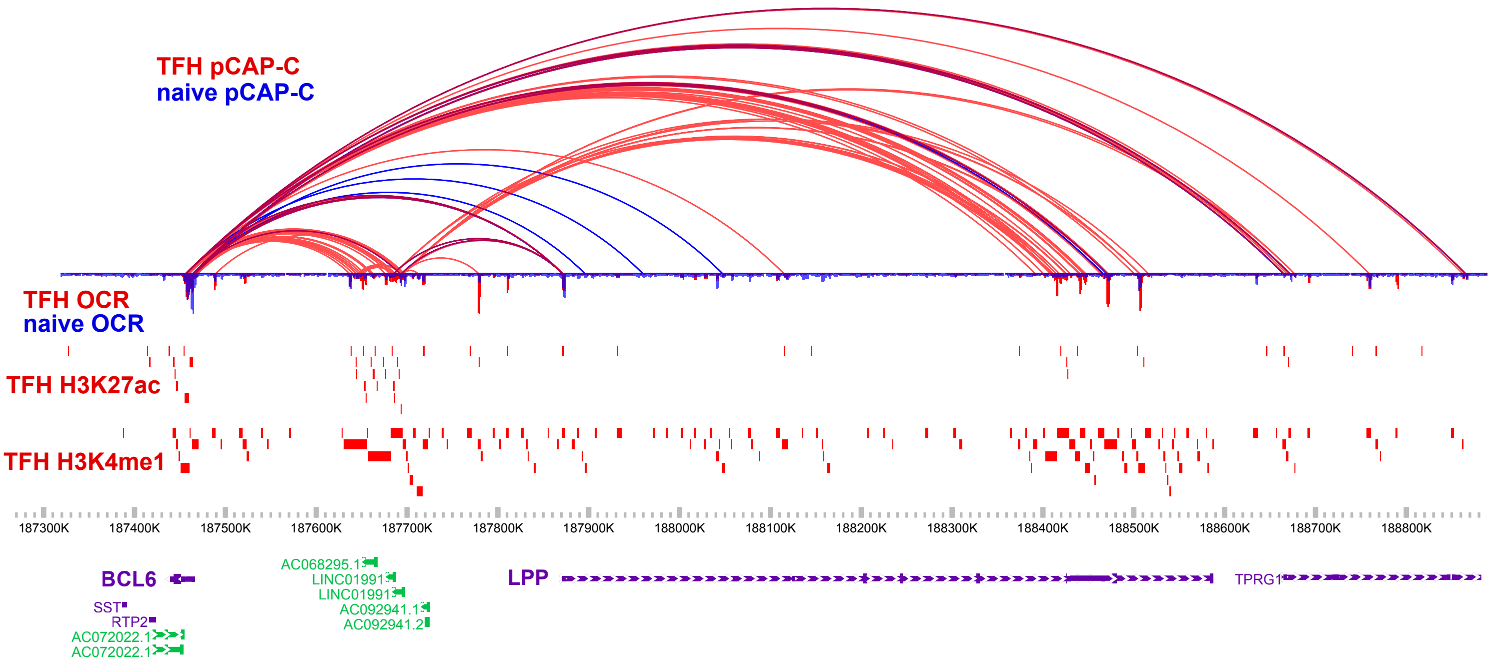

SUPPLEMENTARY FIGURE 11. 3D epigenomic map of promoter-Capture-C, ATAC-seq, H3K27ac, and H3K4me1 in the BCL6-LPP region in naïve (blue) and TFH cells (red).

SUPPLEMENTARY FIGURE 12

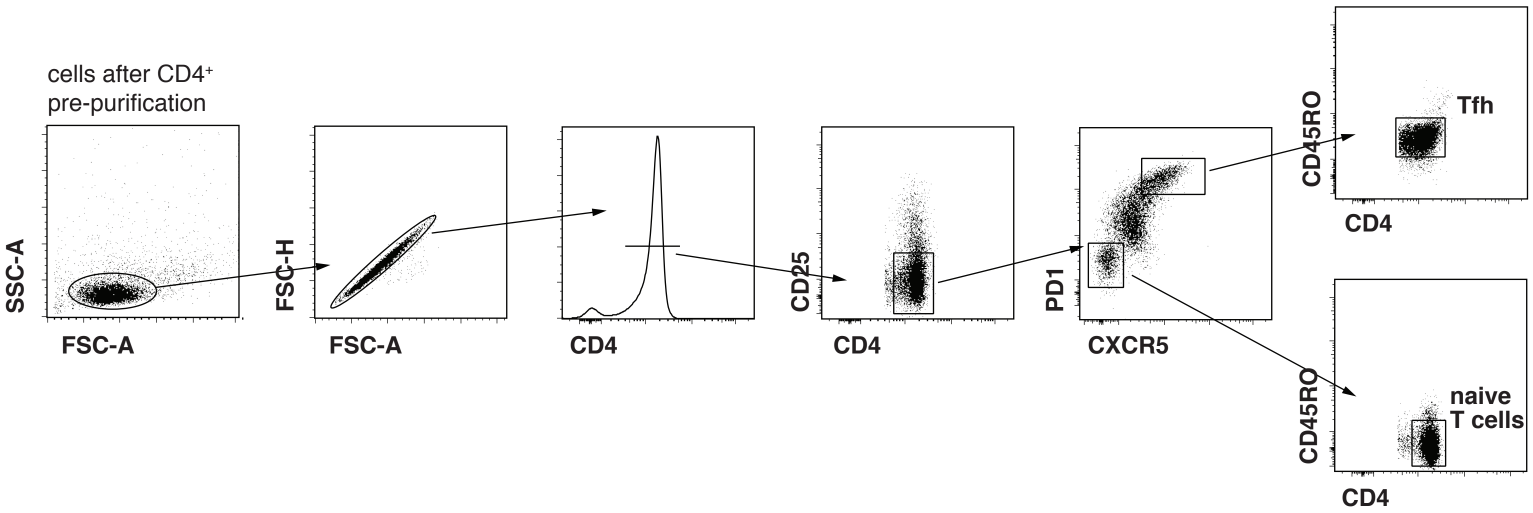

SUPPLEMENTARY FIGURE 12. Gating strategy for sorting of tonsillar naive CD4+ T cells and follicular helper T cells used for generation of promoter Capture-C, ATAC-seq, and transcriptomic data. Cell preparation and staining details are listed in the Methods section.

Supplementary Table 1. LDSC analysis.

disease_atacSet	Prop_SNPs	Prop_h2	Prop_h2_std	Enrichment	Enrichment_std	Enrichment_p
SLE_TFH_atac	0.017535355	0.271554626	0.095119975	15.48612082	5.424468145	0.003505724
SLE_naive_atac	0.017643512	0.247419471	0.102033394	14.02325559	5.783054788	0.011160528
SLE_TFH_iOCR	0.007115549	0.144754605	0.071080947	20.34341972	9.989523552	0.013687843
SLE_naive_iOCR	0.006899405	0.106275957	0.066712335	15.40364091	9.669288139	0.066323836

Supplementary Table 2. CHiCAGO summary.

	Naïve			TFH		
	1frag	4frag	merge	1frag	4frag	merge
Number of bait fragments with interactions	26366 (71.86%)	26861 (78.19%)	48767 (68.64%)	25401 (69.23%)	25846 (75.23%)	46666 (65.69%)
Number of interactions	143323	195464	255238	118410	177653	224263
Number of trans interactions	791 (0.55%)	766 (0.39%)	1026 (0.40%)	1480 (1.25%)	883 (0.50%)	1747 (0.78%)
Number bait-to-bait interactions	19290 (13.46%)	41715 (21.34%)	48995 (19.20%)	17784 (15.02%)	41180 (23.18%)	47299 (21.09%)
Median bait-OE distance for cis interactions (including bait-to-bait)	22,735	93,332.50	57,263.00	18,544	87,034.50	54,270.50
Median number of interactions per bait fragment (including bait-to-bait)	2	3		2	3	
Median number of cis-interactions per bait fragment (including bait-to-bait)	2	3		2	3	
Number of unique non-bait OE	101603	96638		83295	86393	
Median number of interacting bait fragments per non-bait OE	1	1		1	1	
Number of non-bait OE interacting with a single bait fragment	85790 (84.44%)	65711 (68.00%)		70825 (85.03%)	59167 (68.49%)	
Number of non-bait OE interacting with >=4 bait fragments	1426 (1.40%)	6201 (6.42%)		1024 (1.23%)	5398 (6.25%)	

Supplementary Table 3. Interaction summary.

		TFH	naïve	Ref
iOCR number		25,813	24,470	31,404
	promoter-proximal region	14,617	14,275	16,295
	promoter distal region	11,196	10,195	15,109
Interaction number		52,958	48,496	71,137
	promoter-proximal region to promoter-proximal region	21,484	19,332	30,815
	promoter-proximal region to promoter-distal region	31,474	29,164	40,322
median distance		105,074	110,632	117,496
	promoter-proximal region to promoter-proximal region	104,022	110,620	112,492
	promoter-proximal region to promoter-distal region	106,484	110,660	122,972
Transcript number		74,504	73,796	79,330
	promoter-proximal region to promoter-proximal region	73,460	72,472	78,357
	promoter-proximal region to promoter-distal region	32,336	31,528	39,404
Gene number		17,324	17,143	18,669
	promoter-proximal region to promoter-proximal region	16,973	16,744	18,310
	promoter-proximal region to promoter-distal region	7,995	7,746	9,724
