# Supplementary material for: Mapping effector genes at lupus GWAS loci using promoter Capture-C in follicular helper T cells
Source: Nat Commun. 2020 Jul 3;11:3294. doi: 10.1038/s41467-020-17089-5 (PMC7335045; doi:10.1038/s41467-020-17089-5)
Supplement: Supplementary file 3 — Descriptions of Additional Supplementary Files [file 41467_2020_17089_MOESM3_ESM.docx]

**Descriptions of Additional Supplementary Files**

**File name:** Supplementary Dataset 1

**Description:** Reference ATAC-seq peaks in naive and TFH cells

**File name:** Supplementary Dataset 2

**Description:** Microarray-based gene expression analysis

**File name:** Supplementary Dataset 3

**Description:** OCR harboring SLE proxy SNPs

**File name:** Supplementary Dataset 4

**Description:** OCR harboring SLE proxy SNPs

**File name:** Supplementary Dataset 5

**Description:** HiCUP summary
