# Supplementary material for: Mapping effector genes at lupus GWAS loci using promoter Capture-C in follicular helper T cells
Source: Nat Commun. 2020 Jul 3;11:3294. doi: 10.1038/s41467-020-17089-5 (PMC7335045; doi:10.1038/s41467-020-17089-5)
Supplement: Supplementary file 9 — Reporting Summary [file 41467_2020_17089_MOESM9_ESM.pdf]

nature research

Corresponding author(s): Andrew Wells, Struan Grant

Last updated by author(s): 5-8-2020

Reporting Summary

Nature Research wishes to improve the reproducibility of the work that we publish. This form provides structure for consistency and transparency in reporting. For further information on Nature Research policies, see Authors & Referees and the Editorial Policy Checklist .

Statistics

For all statistical analyses, confirm that the following items are present in the figure legend, table legend, main text, or Methods section.

n/a	Confirmed	
☐	☒	The exact sample size (*n*) for each experimental group/condition, given as a discrete number and unit of measurement
☐	☒	A statement on whether measurements were taken from distinct samples or whether the same sample was measured repeatedly
☐	☒	The statistical test(s) used AND whether they are one- or two-sided<br>*Only common tests should be described solely by name; describe more complex techniques in the Methods section.*
☐	☒	A description of all covariates tested
☐	☒	A description of any assumptions or corrections, such as tests of normality and adjustment for multiple comparisons
☐	☒	A full description of the statistical parameters including central tendency (e.g. means) or other basic estimates (e.g. regression coefficient) AND variation (e.g. standard deviation) or associated estimates of uncertainty (e.g. confidence intervals)
☐	☒	For null hypothesis testing, the test statistic (e.g. *F*, *t*, *r*) with confidence intervals, effect sizes, degrees of freedom and *P* value noted<br>*Give P values as exact values whenever suitable.*
☒	☐	For Bayesian analysis, information on the choice of priors and Markov chain Monte Carlo settings
☒	☐	For hierarchical and complex designs, identification of the appropriate level for tests and full reporting of outcomes
☐	☒	Estimates of effect sizes (e.g. Cohen's *d*, Pearson's *r*), indicating how they were calculated

Our web collection on statistics for biologists contains articles on many of the points above.

Software and code

Policy information about availability of computer code

Data collection	ENCODE ATAC-seq pipeline (https://www.encodeproject.org/atac-seq/), bedtools (v2.25.0), R package csaw (v 1.8.1), edgeR (v 3.16.5), limmaVoom (v 3.30.13), HICUP pipeline (v0.5.9), CHiCAGO (v1.1.8), Transcriptome Analysis Console v 4.0, ChromHMM (v1.17), Ingenuity pathway analysis (IPA, QIAGEN), Flowjo (v10)
Data analysis	TFH and naïve ATAC-seq peaks were called using the ENCODE ATAC-seq pipeline (https://www.encodeproject.org/atac-seq/). Briefly, pair-end reads from three biological replicates for each cell type were aligned to hg19 genome using bowtie2, and duplicate reads were removed from the alignment. Narrow peaks were called independently for each replicate using macs2 (-p 0.01 --nomodel --shift -75 --extsize 150 -B --SPMR --keep-dup all --call-summits) and ENCODE blacklist regions (ENCSR636HFF) were removed from peaks in individual replicates. Peaks from all replicates were merged by bedtools (v2.25.0) within each cell type and the merged peaks present in less than two biological replicates were removed from further analysis. Finally, ATAC-seq peaks from both cell types were merged to obtain reference open chromatin regions. To determine whether an OCR is present in TFH and/or naïve cells, we first intersected peaks identified from individual replicates in each cell type with reference OCRs. If any peaks from at least one replicate overlapped with a given reference OCR, we consider that region is open in the originating cell type. Quantitative comparisons of TFH and naïve open chromatin landscapes were performed by evaluating read count differences against the reference OCR set. De-duplicated read counts for OCR were calculated for each library and normalized against background (10K bins of genome) using the R package csaw (v 1.8.1). OCR peaks with less than 1.5 CPM (4.5 ~ 7.5 reads) support in the top 50% of samples were removed from further differential analysis. Differential analysis was performed independently using edgeR (v 3.16.5) and limmaVoom (v 3.30.13). Differential OCR between cell types were called if FDR<0.05 and absolute log2 fold change >1 in both methods. For Capture-C analysis, paired-end reads from three biological replicates for naïve and follicular helper T cells were pre-processed using the HICUP pipeline (v0.5.9) {Wingett:2015}, with bowtie2 as aligner and hg19 as the reference genome. We were able to detect non-hybrid reads from all targeted promoters, validating the success of the promoter capture procedure. Significant promoter interactions at 1-DpnII fragment resolution were called using CHiCAGO (v1.1.8) {Cairns:2016} with default parameters except for binsize set to 2500. Significant interactions at 4-DpnII fragment resolution were also called using CHiCAGO with artificial .baitmap and .rmap files in which DpnII fragments were concatenated in silico into 4 consecutive fragments using default parameters except for removeAdjacent set to False. The significant interactions (CHiCAGO score > 5) from both 1-fragment and 4-fragment resolutions were exported in .ibed format and merged into a single file using custom a

PERL script to remove redundant interactions and to keep the max CHiCAGO score for each interaction. For microarray analysis, data were pre-processed (RMA normalization), and analyzed for differential expression (DE) using Transcriptome Analysis Console v 4.0 with a false discovery rate (FDR) threshold of 0.05 and a fold-change (FC) threshold of 2.

For manuscripts utilizing custom algorithms or software that are central to the research but not yet described in published literature, software must be made available to editors/reviewers. We strongly encourage code deposition in a community repository (e.g. GitHub). See the Nature Research guidelines for submitting code & software for further information.

Data

Policy information about availability of data

All manuscripts must include a data availability statement. This statement should provide the following information, where applicable:
- Accession codes, unique identifiers, or web links for publicly available datasets
- A list of figures that have associated raw data
- A description of any restrictions on data availability

Our data are publicly available from ArrayExpress (https://www.ebi.ac.uk/arrayexpress/) with accession numbers E-MTAB-6621 (promoter-Capture-C), E-MTAB-6617 (ATAC-seq), and E-MTAB-6637(expression microarray) respectively.

Field-specific reporting

Please select the one below that is the best fit for your research. If you are not sure, read the appropriate sections before making your selection.

[x] Life sciences [ ] Behavioural & social sciences [ ] Ecological, evolutionary & environmental sciences

For a reference copy of the document with all sections, see nature.com/documents/nr-reporting-summary-flat.pdf

Life sciences study design

All studies must disclose on these points even when the disclosure is negative.

Sample size	An N of 3 provides the statistical power for all algorithms to call ATAC-seq peaks, differential gene expression, and promoter interaction calls.
Data exclusions	No data were excluded from the study
Replication	An N of 3 donor replicates provides the statistical power for all algorithms to call ATAC-seq peaks, differential gene expression, and promoter interaction calls, and all replication attempts were successful.
Randomization	The study design requires tonsil samples available same day and does not control donor characteristics. All samples are processed together to avoid downstream batch effects in the genome-scale data sets
Blinding	All donor samples and sorted populations were processed at the same time. Samples were processed for ATAC-seq and promoter Capture-C in a blinded fashion. Downstream data analysis pipelines required knowledge of which samples were paired with each donor, but the analysis process was blinded to the identity of the sample types (e.g., naive vs. TFH) within each donor set.

Reporting for specific materials, systems and methods

We require information from authors about some types of materials, experimental systems and methods used in many studies. Here, indicate whether each material, system or method listed is relevant to your study. If you are not sure if a list item applies to your research, read the appropriate section before selecting a response.

Materials & experimental systems

n/a	Involved in the study	
[ ]	[x]	Antibodies
[ ]	[x]	Eukaryotic cell lines
[x]	[ ]	Palaeontology
[x]	[ ]	Animals and other organisms
[ ]	[x]	Human research participants
[x]	[ ]	Clinical data

Methods

n/a	Involved in the study	
[x]	[ ]	ChIP-seq
[ ]	[x]	Flow cytometry
[x]	[ ]	MRI-based neuroimaging

Antibodies

Antibodies used	Anti-human APC-BCL-6 (Biolegend, cat # 358506, 5 ul/test) after treatment with human recombinant IFNg (5 ng/mL, R&D Systems, cat # 285-IF) overnight and stimulation with PMA (30 ng/mL, cat # P-8139) and ionomycin (1 uM, Sigma-Aldrich, cat # I-0634) for 4-6 hrs. Expression of Ikaros and CXCR5 by targeted Jurkat lines was also assessed by flow cytometry using anti-human APC-CXCR5 (Biolegend, cat # 356908, 5 ul/test) and anti-human PE-Ikaros (BD Biosciences, cat # 564476, 5 ul/test). Biolegend antibodies Anti-human PE/Dazzle 594-CXCR5 (5 ul/test) cat # 356928 and Anti-human Brilliant Violet 421-PD-1 (5 ul/

	test) cat # 329920 were used for the human T cells staining. Biolegend antibodies CD4 APC-Cy7 (1/100 dilution, cat. 317418), CD45RO Pac Blue (1/50, cat. 304216), CD25 PE (1/25, cat. 302606), CXCR5 APC (1/10, cat. 356908), PD1 PeCy7 (1/50, cat. 329918) were used for tonsillar T cell sorting.
Validation	All antibodies against human CD markers are titrated and used routinely in the laboratory and are validated using primary human lymphocytes that have predicted marker expression patterns (positive controls), flourescence-minus-one (FMO) controls, and by staining of human lymphocytes known not to express the target antigens (negative controls).

Eukaryotic cell lines

Policy information about cell lines

Cell line source(s)	Jurkat and 293T from ATCC
Authentication	Stimulation-induced IL-2 and BCL-6 expression by Jurkat cells was confirmed
Mycoplasma contamination	Cells are mycoplasma-free and only used for ~6 passages after thawed from cryopreserved stocks
Commonly misidentified lines (See ICLAC register)	No commonly misidentified cell lines were used in this study.

Human research participants

Policy information about studies involving human research participants

Population characteristics	tonsil donors (n=3, age range 2-16 years) are pediatric patients undergoing tonsillectomy at The Children's Hospital of Philadelphia for treatment of sleep apnea
Recruitment	tonsil donors are pediatric patients undergoing tonsillectomy at The Children's Hospital of Philadelphia for treatment of sleep apnea. Use of discarded tissue does not require recruitment or consent.
Ethics oversight	The Children's Hospital of Philadelphia Institutional Review Board approved this use of de-identified discarded tissue categorized as non-human subject research

Note that full information on the approval of the study protocol must also be provided in the manuscript.

Flow Cytometry

Plots

Confirm that:

- [x] The axis labels state the marker and fluorochrome used (e.g. CD4-FITC).
- [x] The axis scales are clearly visible. Include numbers along axes only for bottom left plot of group (a 'group' is an analysis of identical markers).
- [x] All plots are contour plots with outliers or pseudocolor plots.
- [x] A numerical value for number of cells or percentage (with statistics) is provided.

Methodology

Sample preparation	Fresh tonsils were obtained from immune-competent children (n=3) undergoing tonsillectomy to address airway obstruction or a history of recurrent tonsillitis. The mean age of donors was 5.7 years (range 2-16 years) and 50% were male. Tonsillar mononuclear cells were isolated from tissues by mechanical disruption (tonsils were minced and pressed through a 70 micron cell screen) followed by Ficoll-Paque centrifugation. CD19-positive cells were removed (StemCell) and CD4+ T cells were enriched with magnetic beads (Biolegend) prior to sorting.
Instrument	BECKMAN CYTOFLEX ADP, BECKMAN MoFlo Astrios EQ
Software	FLOWJO V10
Cell population abundance	10 million Tonsillar naive CD4+ T cell and CD4+ follicular helper T cell were sorted to >95% purity on a MoFlo Astrios EQ (Beckman Coulter).
Gating strategy	naïve tonsillar T cells (CD4+CD45RO-) and T follicular helper cells (CD4+CD45RO+CD25loCXCR5hiPD1hi) were sorted on a MoFlo Astrios EQ (Beckman Coulter).

- [x] Tick this box to confirm that a figure exemplifying the gating strategy is provided in the Supplementary Information.
